# Sexual Orientation and Exposure to Close Others’ Self-Injurious Thoughts and Behaviors

**DOI:** 10.1001/jamanetworkopen.2025.31182

**Published:** 2025-09-10

**Authors:** Kirsty A. Clark, Seonaid Cleare, Amy M. Brausch, Karen Wetherall, Richard Bränström, Mark L. Hatzenbuehler, John E. Pachankis, Rory C. O’Connor

**Affiliations:** 1 Department of Medicine, Health, and Society, Vanderbilt University, Nashville, Tennessee; 2 Department of Psychology and Human Development, Vanderbilt University, Nashville, Tennessee; 3 Suicidal Behavior Research Lab, School of Health and Wellbeing, University of Glasgow, Glasgow, Scotland; 4 Department of Psychological Sciences, Western Kentucky University, Bowling Green; 5 Department of Clinical Neuroscience, Karolinska Institutet, Stockholm, Sweden; 6 Department of Psychology, Harvard University, Cambridge, Massachusetts; 7 Department of Social and Behavioral Sciences, Yale School of Public Health, New Haven, Connecticut

## Abstract

**Importance:**

Sexual minority young adults experience disproportionately high rates of self-injurious thoughts and behaviors (SITB), including suicidal ideation, suicide attempts, and nonsuicidal self-injury (NSSI). While exposure to close others’ SITB (eg, a friend or family member’s suicide attempt) is a known risk factor for SITB, its role in sexual orientation disparities in SITB remains underexplored.

**Objective:**

To examine whether exposure to close others’ SITB is associated with sexual orientation disparities in SITB.

**Design, Setting, and Participants:**

This cohort study assessed data from 2 nationally representative cohorts: the Swedish Pathways to Longitudinally Understanding Stress (PLUS) study (2023) and the Scottish Well-being Study ([SWS] conducted 2015-2018). The present study uses data only from the final wave of the PLUS study (2023) as this was the only wave that gathered information on exposure to close others’ SITB (n = 1202). The present study also uses data from participants who completed the second through fourth follow-up waves of the SWS, as sexual orientation information was not collected at the first wave (n = 1828).

**Exposures:**

Sexual orientation (heterosexual vs sexual minority) was the primary exposure. Exposure to close others’ SITB, assessed via validated measures, was the primary mediator.

**Main Outcomes and Measures:**

In both cohorts, primary outcomes assessed participants' own SITB. In the PLUS cohort, the primary outcome was suicidal ideation measured using the Suicidal Ideation Attributes Scale (scores range from 0 to 50, with higher scores indicating more severe suicidal ideation). In SWS, there were 5 primary outcomes assessing past-year presence of suicidal ideation, NSSI, NSSI ideation, and suicide attempt as well as a composite indicator of any of these outcomes (none = 0 or any = 1). Mediation analyses estimated associations between sexual minority status and SITB outcomes, mediated by exposure to close others’ SITB. Bootstrap bias–corrected 95% CIs were calculated with 5000 bootstrap samples.

**Results:**

The PLUS cohort included 1202 participants (mean [SD] age, 30.2 [5.1] years; 906 [75.4%] female; 660 [54.9%] heterosexual). The SWS cohort included 1828 participants assessed across 3 waves (mean [SD] age, 26.9 [4.8] years; 1058 [57.9%] female; 1694 [93.1%] heterosexual). Sexual minority participants reported more frequent exposure to close others’ SITB (eg, for suicide attempt: PLUS mean [SD], 0.41 [0.49] for sexual minority vs 0.23 [0.42] for heterosexual; *P* < .001; and SWS mean [SD], 0.28 [0.45] for sexual minority vs 0.14 [0.35] for heterosexual; *P* < .001) and higher SITB rates (eg, suicidal ideation: PLUS mean [SD], 4.67 [8.19] for sexual minority vs 1.97 [5.57] for heterosexual; *P* < .001; and SWS mean [SD], 0.35 [0.48] for sexual minority vs 0.13 [0.33]; *P* < .001) than heterosexual participants. In the PLUS cohort, exposure to close others’ suicidal ideation accounted for 53.3% of the association between sexual orientation and past-month suicidal ideation (bootstrap 95% CI, 36.7%-76.0%). In the SWS cohort, exposure to close others’ SITB (a composite indicator of exposure to close others’ suicide attempt, suicide death, and NSSI) accounted for 46.9% of the association between sexual orientation and past-year NSSI (bootstrap 95% CI, 17.3%-86.3%).

**Conclusions and Relevance:**

Findings from this study of 2 nationally representative cohorts of young adults suggest that exposure to close others’ SITB may partially explain sexual orientation disparities in SITB. Findings underscore the need for further research into psychosocial mechanisms that may propagate SITB risk following exposure to close others’ SITB in vulnerable populations.

## Key Points

**Question:**

Is exposure to close others’ (eg, friends, family members) self-injurious thoughts and behaviors (SITB) associated with sexual orientation disparities in SITB among young adults?

**Findings:**

In this cohort study involving 3030 respondents across 2 nationally representative population-based cohorts, sexual minority young adults reported significantly higher frequency of exposure to close others’ SITB than heterosexual young adults. Mediation analyses indicated that exposure to close others’ SITB accounted for a statistically significant portion of the sexual orientation disparity in SITB.

**Meaning:**

Findings from this cohort study suggest that exposure to close others’ SITB may explain increased SITB risk among sexual minority young adults, warranting further research investigating psychosocial mechanisms underlying SITB transmission within at-risk groups.

## Teaser

This cohort study analyzes data from 2 nationally representative populations to investigate whether exposure to friends’ or family members’ self-injurious thoughts and behaviors is associated with disparities in self-injurious thoughts and behaviors among sexual minority young adults.

## Introduction

Self-injurious thoughts and behaviors (SITB), including suicidal ideation, suicide attempt, and nonsuicidal self-injury (NSSI), are pressing global public health concerns, particularly among young people, for whom suicide ranks as one of the leading causes of mortality worldwide.^1^ A robust body of research has shown that exposure to SITB among close relations—such as hearing a friend or family member express thoughts of suicide or experiencing the suicide death of a loved one—is a substantial risk factor for one’s own SITB and suicide,^2^ suggesting that interpersonal and social dynamics may play critical roles in the transmission of SITB and suicide risk.^3,4^ This risk appears to be particularly relevant during adolescence and young adulthood, when the prevalence of exposure to others’ SITB in one’s social network increases.^5^

Rates of SITB are disproportionately higher among sexual minority individuals (ie, individuals with a nonheterosexual sexual orientation, such as gay, lesbian, and bisexual) than heterosexual individuals, particularly in young adulthood.^6,7^ This disparity is partly attributable to sexual minority people’s increased exposure to stigma-related stressors, such as discrimination and victimization, which are key drivers of suicide risk. Notably, research shows that the association between discrimination and suicide attempts appears strongest in young adulthood.^6^ Emerging evidence further suggests that sexual minority individuals also face elevated exposure to close others’ SITB compared with heterosexual individuals, representing an additional potential pathway to heightened SITB risk.^8^

Two key theories may explain sexual minority people’s heightened exposure to other people’s SITB. First, social network homophily suggests that sexual minority individuals form close-knit networks with others in their community due to shared experiences and the pursuit of social safety amid stigma.^9^ Because SITB rates are higher among sexual minority individuals, these tightly connected networks may inadvertently increase exposure to close others’ SITB. Second, narrative possibilities theory posits that societal discourse portrays SITB and suicide as normative among sexual minority youths because of their presumed vulnerability to distress, which can inadvertently normalize these behaviors for sexual minority young people.^10,11^ Supporting this theory, research shows that sexual minority individuals tend to hold more accepting views of SITB and suicide than heterosexual individuals, and sexual minority individuals with a history of suicide attempts often describe these behaviors as common among their peers.^10,12,13^

Additionally, representative school-based data show that sexual minority youths report living with someone who is depressed, mentally ill, or suicidal at approximately twice the rate of their heterosexual peers.^14^ This finding suggests that sexual minority individuals’ heightened exposure to close others’ SITB may extend beyond peer groups and into families. One possible explanation is that sexual minority individuals report more childhood household instability, including family mental and behavioral health problems, for reasons not yet fully understood.^15^ Taken together, social network structure, common narrative assumptions about sexual minority youths, and family instability and mental health difficulties may contribute to sexual minority individuals’ heightened exposure to close others’ SITB.

The few studies that have examined associations among sexual orientation and exposure to close others’ SITB primarily rely on convenience samples, introducing potential biases arising from selecting groups based on salient characteristics such as sexual orientation or previous SITB history.^16^ Population-based research, especially in diverse national contexts, is needed to account for varying legal, social, and cultural influences on SITB as well as sexual minority social experiences.^17^ The present study addresses these gaps by leveraging 2 population-based cohorts in Northern Europe that are uniquely suited to investigating sexual orientation differences in exposure to close others’ SITB and associations with sexual orientation disparities in SITB.

## Methods

### Participants and Procedures

This cohort study used data from 2 population-based cohort studies: one conducted in Sweden (Pathways to Longitudinally Understanding Stress [PLUS]) and the other in Scotland (Scottish Well-Being Study [SWS]). Details about these studies have been reported elsewhere.^18,19^ Reporting of the present results follows the Strengthening the Reporting of Observational Studies in Epidemiology (STROBE) guideline. For the PLUS cohort, all procedures were approved by the Stockholm Regional Ethical Review Board. For the SWS cohort, ethical approval was obtained from the Department of Psychology Ethics Committee at the University of Stirling and the US Department of Defense, Human Research Protections Office. All participants included in the present study provided written informed consent.

To assemble the PLUS cohort, a follow-back methodology was used to recruit a nationally representative sample of young adults from across Sweden, with a planned 50% of the sample being sexual minority. Specifically, all young adults ages 17 to 34 years who reported a sexual minority identity in the 2015, 2016, and 2018 population-based Swedish National Public Health Survey (n = 2973) were invited to participate alongside a randomly selected comparison sample of heterosexuals (n = 2973). Of those invited, 2222 individuals provided written informed consent and completed the baseline assessment. The PLUS cohort was followed up annually for 5 years from 2019 to 2023. For the present study, we used data from the fifth and final follow-up wave (collected in October 2023), as this was the only wave that gathered information on exposure to close others’ SITB.

To assemble the SWS cohort, quota sampling was used to recruit a nationally representative sample of young adults from across Scotland (N = 3508; 18-34 years of age). Baseline recruitment was carried out between March and December 2013. Participants were invited to take part in follow-up surveys at 12-month (T1; 2015), 24-month (T2; 2016) and 36-month (T3; 2017-2018) follow-up. For the present study, we included data from participants who contributed data in T1, T2, and T3. The SWS baseline assessment (conducted at T0) did not assess sexual orientation and thus was not used.

Ethnicity was not reported in PLUS but was self-reported in the SWS to more fully characterize the cohort. Categories included Black, Chinese or other Asian, European White, South Asian, or other ethnicity. Due to Scotland's low level of ethnic diversity, subgroups comprising other ethnicities were not assessed.

### Measures

#### Sexual Orientation

In the PLUS cohort, participants answered, “Which of the following best represents how you think of yourself?” with 5 possible response options. Individuals selecting “Straight, that is, not lesbian or gay” were categorized as heterosexual (0), while all others (“lesbian or gay,” “bisexual,” “something else,” “I don’t know”) were classified as sexual minority (1). In the SWS cohort, participants responded to a similar question with 8 possible response options. Those selecting “heterosexual or straight” were categorized as heterosexual (0), with all others classified as sexual minority (1).

#### Exposure to Close Others’ SITB

In the PLUS cohort, participants answered 2 yes or no questions assessing exposure to a close other’s suicide attempt or death,^20^ which were assessed separately and also used to form a composite indicator (0 = no exposure, 1 = any exposure). Participants also completed the Exposure to Suicidal Communication subscale of the Suicidal Behavior Exposure Scale (Cronbach α = 0.84), a 4-item scale assessing frequency of exposure to others’ suicidal ideation (sum score, 0-16, with higher numbers indicating greater frequency of exposure to others' suicidal ideation).^21^ In the SWS cohort, exposure to close others’ SITB was assessed at T2 using 3 separate items from the List of Threatening Experiences questionnaire^22^ assessing exposure to a friend or family member’s NSSI, suicide attempt, or suicide death. Responses were categorized as no exposure (0) or any exposure (1), and a composite indicator of overall exposure to close others’ SITB was also created.

#### SITB Outcomes

In the PLUS cohort, past-month suicidal ideation was measured via the 5-item Suicide Ideation Attributes Scale (SIDAS), with scores ranging from 0 to 50, higher scores indicating greater severity (Cronbach α = 0.89).^23^ In the SWS cohort, past-year suicidal ideation, NSSI, NSSI ideation, and suicide attempt were assessed via 4 binary (yes or no) items from the Adult Psychiatric Morbidity Survey.^24^ Each outcome was assessed separately, and a composite indicator reflecting no (0) vs any (1) SITB endorsement was also created.

#### Covariates

Analyses were adjusted for age and sex assigned at birth given associations between these demographic factors and sexual orientation, exposure to others’ SITB, and SITB.^25,26,27^ Supplemental analyses additionally controlled for stigma-related stress: discrimination (PLUS) via the Everyday Discrimination Scale (Cronbach α = 0.88)^28^ and victimization (SWS) via a dichotomized item on the List of Threatening Experiences questionnaire for exposure to bullying or victimization.

### Supplemental Analyses

We conducted 3 supplemental analyses. First, we calculated descriptive statistics (means and SDs) for all SITB variables, stratified by sexual orientation and sex. Among males and females separately, sexual orientation group differences (reference group: heterosexual) were computed with linear or logistic regression models for continuous or binary SITB variables, respectively. This analysis was restricted to the PLUS study due to its adequate sample size for disaggregating by sexual orientation and sex. Second, we reran all main mediation models (in PLUS and the SWS), including exposure to stigma-related stress (measured by discrimination in PLUS and victimization in the SWS) as a covariate. Third, we reran the main mediation models in the PLUS study using a binary outcome of suicidal ideation (any vs none) instead of the continuous SIDAS score to account for the nonnormal distribution of the SIDAS score in a more interpretable manner.

### Statistical Analysis

In both cohorts, analyses were conducted in 3 sequential steps using SAS, version 9.4 (SAS Institute Inc), and a 2-sided *P* < .05 was considered statistically significant. First, descriptive statistics, including means and SDs, were calculated for all variables of interest. We also calculated Pearson correlations to assess unadjusted associations among all variables of interest. In the PLUS cohort, we assessed the distributional properties of the SIDAS, finding that it demonstrated a nonnormal distribution (skewness = 2.85; kurtosis = 8.40). To address this nonnormal distribution, a log transformation was applied, resulting in a distribution approximating normality (skewness = 1.34; kurtosis = 0.41). Because the pattern of results was consistent across both the transformed and untransformed scores, we present findings using the untransformed SIDAS for ease of interpretation.

Second, data were stratified by sexual orientation (sexual minority vs heterosexual) and means and SDs of SITB variables (mediators and outcomes) were computed within each group. Sexual orientation group differences were assessed using independent *t* tests. Third, mediation analyses were performed within a potential outcomes framework using the CAUSALMED procedure, which estimates causal mediation effects from observational data, allows for exposure-mediator interaction, decomposes the total effect into direct and indirect effects, and handles dichotomous mediators.^29^ The CAUSALMED procedure was used to estimate the total effect (TE), natural direct effect (NDE), and natural indirect effect (NIE) of sexual minority status on SITB outcomes, mediated by exposure to close others’ SITB.^29^ The TE reflects the sum of the NDE and NIE. The percentage of mediation was calculated by the formula (NIE/TE) × 100. Bootstrap bias–corrected 95% CIs were calculated with 5000 bootstrap samples. Interaction terms were included to allow exposure to close others’ SITB to vary by sexual orientation.^30^

In the PLUS cohort, 2 separate cross-sectional mediation models were tested. In both models, sexual orientation was the exposure, and past-month suicidal ideation (measured by the SIDAS score) was the outcome. In the first model, the mediator was the composite binary indicator of exposure to close others’ suicide attempt or death. In the second model, the mediator was the continuous subscale score measuring exposure to close others’ suicidal ideation. In the SWS cohort, 5 separate mediation models were tested. In all models, sexual orientation assessed at T1 served as the exposure, and the composite binary indicator of exposure to close others’ SITB, assessed at T2, was the mediator. The outcomes, assessed at T3, varied across the models and included past-year suicidal ideation (model 1), past-year NSSI (model 2), past-year NSSI ideation (model 3), past-year suicide attempt (model 4), and the composite indicator of any past-year SITB (model 5).

## Results

### Descriptive Statistics and Pearson Correlations

Demographic characteristics were comparable between the PLUS (n = 1202) and SWS (n = 1828) cohorts, with both samples predominantly assigned female sex at birth (PLUS: 906 [75.4%] female and 296 [24.6%] male; SWS: 1058 [57.9%] female and 770 [42.1%] male). Approximately 1 in 5 participants were married or in a civil partnership (PLUS, 264 [22.0%]; SWS, 409 [22.4%]), and about half were engaged in full-time employment (PLUS, 664 [55.2%]; SWS, 903 [50.6%]) (Table 1). The mean (SD) age was 30.2 (5.1) years in the PLUS cohort and 26.9 (4.8) years in the SWS cohort. In the SWS cohort, self-reported ethnicity was European White for 1741 individuals (96.1%) and ethnic minority for 71 individuals (3.9%), which included Black, Chinese or other Asian, South Asian, or other ethnicity. The primary distinction between the 2 samples was the proportion of sexual minority participants. In the PLUS cohort, 542 participants (45.1%) identified as sexual minority individuals, compared with only 125 participants (6.8%) in the SWS cohort, given the planned composition of the PLUS study to include equal numbers of sexual minority and heterosexual participants.

**Table 1.** Sample Characteristics of Both Cohorts

Variable	Respondents, No. (%)	
	PLUS (n = 1202)^a^	SWS (n = 1828)^b^
Age, mean (SD), y	30.2 (5.1)	26.9 (4.8)
Sex		
Female	906 (75.4)	1058 (57.9)
Male	296 (24.6)	770 (42.1)
Sexual orientation		
Heterosexual (straight)	660 (54.9)	1694 (93.1)
Lesbian or gay	116 (9.7)	64 (3.5)
Bisexual	331 (27.5)	51 (2.8)
Something else or not sure	95 (7.9)	10 (0.6)
Immigration status		
Born in Sweden	1103 (91.8)	NA
Migrated to Sweden	99 (8.2)	NA
Ethnicity^c^		
European White	NA	1741 (96.1)
Ethnic minority	NA	71 (3.9)
Marital status		
Married or civil partnership	264 (22.0)	409 (22.4)
Not married	938 (78.0)	1419 (77.6)
Economic engagement		
Full time work (≥40 h per wk)	664 (55.2)^a^	903 (50.6)
Part time work (<40 h per wk)	247 (20.6)	302 (16.9)
Unemployed, full-time student	164 (13.6)	284 (15.9)
Unemployed, other	126 (10.5)	294 (16.5)

Abbreviations: NA, not assessed; PLUS, Pathways to Longitudinally Understanding Stress; SWS, Scottish Well-Being Study.

^a^ Variables in the PLUS study with any missing data included age (n = 1196) and economic engagement (n = 1201).

^b^ Variables in the SWS cohort with any missing data included sexual orientation (n = 1819), ethnic minority (N = 1812), and economic engagement (n = 1783).

^c^ Ethnic minority is a composite variable including persons who reported being Black, Chinese or other Asian ethnicity, South Asian, or other ethnicity.

Pearson correlations among all variables from the PLUS and SWS cohorts are detailed in eTables 1 and 2 in Supplement 1, respectively. In both cohorts, sexual minority status was negatively associated with age and positively associated with discrimination or victimization and all SITB variables. Sexual orientation group differences across all SITB variables are presented in Table 2 and Table 3. Compared with heterosexual respondents, sexual minority respondents reported significantly greater exposure to close others’ suicidal ideation (PLUS: mean [SD], 5.39 [3.22] for sexual minority vs 3.66 [2.92] for heterosexual; *P* < .001), suicide attempt (PLUS: mean [SD], 0.41 [0.49] for sexual minority vs 0.23 [0.42] for heterosexual; *P* < .001; SWS: mean [SD], 0.28 [0.45] for sexual minority vs 0.14 [0.35] for heterosexual; *P* < .001), suicide death (PLUS: mean [SD], 0.25 [0.43] for sexual minority vs 0.19 [0.40] for heterosexual; *P* = .007; SWS: mean [SD], 0.20 [0.40] for sexual minority vs 0.10 [0.30] for heterosexual; *P* < .001), or NSSI (SWS: mean [SD], 0.30 [0.46] for sexual minority vs 0.14 [0.35] for heterosexual; *P* < .001). Furthermore, sexual minority participants reported significantly higher levels of suicidal ideation (PLUS: mean [SD], 4.67 [8.19] for sexual minority vs 1.97 [5.57] for heterosexual, *P* < .001; SWS: mean [SD], 0.35 [0.48] for sexual minority vs 0.13 [0.33] for heterosexual, *P* < .001), and in SWS for NSSI (mean [SD], 0.14 [0.35] for sexual minority vs 0.05 [0.23] for heterosexual, *P* < .001), NSSI ideation (mean [SD], 0.25 [0.44] for sexual minority vs 0.08 [0.27] for heterosexual, *P* < .001), and suicide attempt (mean [SD], 0.07 [0.26] for sexual minority vs 0.01 (0.10] for heterosexual, *P* < .001) compared with heterosexual respondents.

**Table 2.** Sexual Orientation Differences in Self-Injurious Thoughts and Behaviors Variables, Pathways to Longitudinally Understanding Stress Cohort

Variable	Sexual minority		Heterosexual		*P* value
	No.	Mean (SD)	No.	Mean (SD)	
Exposure to close others’ suicide attempt^a^	541	0.41 (0.49)	660	0.23 (0.42)	<.001
Exposure to close others’ suicide death^a^	541	0.25 (0.43)	660	0.19 (0.40)	.007
Composite exposure to close others’ suicide attempt or suicide death^a^	542	0.52 (0.50)	660	0.35 (0.48)	<.001
Exposure to close others’ suicidal ideation^b^	542	5.39 (3.22)	660	3.66 (2.92)	<.001
Past-month suicidal ideation^c^	541	4.67 (8.19)	659	1.97 (5.57)	<.001

^a^ 0 = no (unexposed); 1 = yes (exposed).

^b^ Exposure to close others’ suicidal ideation measured through the Suicidal Behavior Exposure Suicidal Communication subscale (scores range from 0 to 16, with higher scores indicating greater frequency of exposure to others' suicidal ideation).

^c^ Past-month suicidal ideation measured through the Suicide Ideation Attributes Scale, with scores ranging from 0 to 50, and higher scores indicating greater severity.

**Table 3.** Sexual Orientation Differences in SITB Variables, Scottish Well-Being Study Cohort

Variables	Respondents at Time 1^a^				*P* value
	Sexual minority		Heterosexual		
	No.	Mean (SD)	No.	Mean (SD)	
Time 2 variables (mediators)^b^					
Exposure to close others’ suicide attempt	123	0.28 (0.45)	1675	0.14 (0.35)	<.001
Exposure to close others’ suicide death	122	0.20 (0.40)	1671	0.10 (0.30)	<.001
Exposure to close others’ NSSI	122	0.30 (0.46)	1677	0.14 (0.35)	<.001
Composite exposure to close others’ suicide attempt or suicide death or NSSI	123	0.44 (0.50)	1680	0.23 (0.42)	<.001
Time 3 variables (outcomes)^c^					
Past-year suicidal ideation	122	0.35 (0.48)	1690	0.13 (0.33)	<.001
Past-year NSSI	122	0.14 (0.35)	1681	0.05 (0.23)	<.001
Past-year NSSI ideation	123	0.25 (0.44)	1678	0.08 (0.27)	<.001
Past-year suicide attempt	122	0.07 (0.26)	1682	0.01 (0.10)	<.001
Composite indicator of any past-year SITB	123	0.46 (0.50)	1691	0.16 (0.37)	<.001

Abbreviations: NSSI, nonsuicidal self-injury; SITB, self-injurious thoughts and behaviors.

^a^ Time 1 = baseline; all variables coded as 0 = no or 1 = yes.

^b^ Time 2 = 12-month follow-up; all variables coded as 0 = no or 1 = yes.

^c^ Time 3 = 24-month follow-up; all variables coded as 0 = no or 1 = yes.

### Mediation Analyses

**Figure 1.**
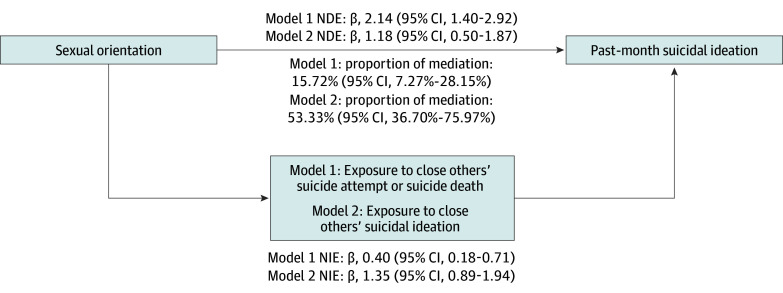
Mediation Models Depicting Direct and Indirect Effect Estimates of Sexual Orientation to Suicidal Ideation Via Exposure to Close Others’ Self-Injurious Thoughts and Behaviors, Pathways to Longitudinally Understanding Stress Cohort Natural direct effect (NDE) and natural indirect effect (NIE) estimates reported as β (bias-corrected bootstrap 95% CI). Proportion of mediation reported as percentage mediated (bias-corrected bootstrap 95% CI). Model 1 mediator: exposure to close others’ suicide attempt/suicide death (exposed = 1; unexposed = 0). Model 2 mediator: exposure to close others’ suicidal ideation (Suicidal Behavior Exposure Suicidal Communication subscale score). Both models: exposure = sexual orientation (sexual minority = 1; heterosexual = 0), outcome = past-month suicidal ideation (Suicidal Ideation Attributes Scale score). Both models adjusted for age (in years) and sex assigned at birth (male, female).

**Figure 2.**
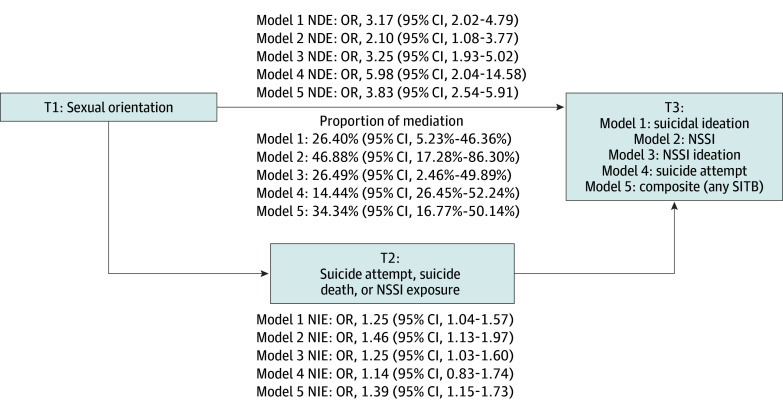
Mediation Models Depicting Direct and Indirect Effect Estimates of Sexual Orientation to Self-Injurious Thoughts and Behaviors (SITB) Via Exposure to Close Others’ SITB, Scottish Well-Being Study Cohort Natural direct effect (NDE) and natural indirect effect (NIE) estimates reported as odds ratio (bias-corrected bootstrap 95% CI). Proportion of mediation reported as percentage mediated (bias-corrected bootstrap 95% CI). T1 represents time 1; T2, time 2 (12-month follow-up); and T3, time 3 (24-month follow-up). Model 1 outcome of past-year suicidal ideation (1 = any; 0 = none); model 2 outcome = past-year SITB engagement (1 = any; 0 = none); model 3 outcome = past-year SITB ideation (1 = any; 0 = none); model 4 outcome = past-year suicide attempt (1 = any; 0 = none); and model 5 outcome = past-year SITB ideation or behavior (1 = any; 0 = none). For all models, the exposure is sexual orientation (sexual minority = 1; heterosexual = 0) and the mediator is suicide or SITB exposure (exposed = 1; unexposed = 0). Models are adjusted for age (in years) and sex assigned at birth (male, female). NSSI indicates nonsuicidal self-injury; OR, odds ratio.

Results from the primary mediation analyses are presented in Figures 1 and 2. In the PLUS study (Figure 1), across both mediation models, the NDE of sexual orientation on past-month suicidal ideation and the NIE through exposure to close others’ suicide attempt or suicide death (model 1: NDE β, 2.14 [95% CI, 1.40-2.92]; and NIE β, 0.40 [95% CI, 0.18-0.71]) and suicidal ideation (model 2: NDE β, 1.18 [95% CI, 0.50-1.87]; and NIE β, 0.40 [95% CI, 0.18-0.71]) were statistically significant. Proportion of mediation analyses showed that exposure to close others’ suicidal ideation accounted for more than half of the association between sexual orientation and past-month suicidal ideation (53.3% [bootstrap 95% CI, 36.7%–76.0%]).

In the SWS cohort (Figure 2), across all models, the NDE of minority sexual orientation (at T1) on the SITB outcomes (at T3) were statistically significant (eg, model 1 NDE: odds ratio, 3.17 [95% CI, 2.02-4.79]). All NIE were statistically significant for all SITB outcomes except past-year suicide attempt (eg, model 1 NIE: odds ratio, 1.25 [95% CI, 1.04-1.57). Analyses of the proportion mediated indicated that exposure to close others’ SITB (at T2) accounted for the largest mediated effect in the association between sexual orientation and past-year NSSI (46.9% [bootstrap 95% CI, 17.3%-86.3%]), followed by the composite outcome of any past-year SITB (34.3% [bootstrap 95% CI, 16.8%-50.1%]), when compared with the other SITB outcomes.

### Supplemental Analyses

First, when disaggregating SITB variables by sex and sexual orientation in the PLUS study, distinct patterns emerged. Exposure to close others’ suicidal ideation was significantly higher in all minority sexual orientation groups (lesbian or gay, bisexual, and something else or I don’t know) compared with heterosexual, regardless of sex. However, among male respondents, there were no significant differences in exposure to close others’ suicide attempt or death by sexual orientation. Among female respondents, exposure to close others’ suicide attempt was increased among all minority sexual orientation groups, whereas exposure to close others’ suicide death was increased only among bisexual respondents. Across both sexes, past-month suicidal ideation was significantly increased among bisexual respondents and persons identifying as something else or I don’t know, but not among lesbian or gay respondents, compared with heterosexual respondents (eTable 3 in Supplement 1). These post hoc analyses should be interpreted with some caution given the small sample sizes when disaggregating by sex and sexual orientation.

Second, when primary mediation models included discrimination (PLUS) and victimization (SWS) as covariates, results remained largely consistent with the primary analyses, although some estimates and bootstrap 95% CIs were slightly attenuated. Thus, the findings held even after accounting for stigma-related stress (eFigures 1 and 2 in Supplement 1).

Third, in mediation models using a binary outcome for past-month suicidal ideation in the PLUS study, results were largely consistent with the primary analyses. However, the proportions of mediation estimates were moderately attenuated (eFigure 3 in Supplement 1).

## Discussion

This cohort study provides novel insights into the role of exposure to close others’ SITB in explaining sexual orientation disparities in SITB during young adulthood, a developmental period of heightened risk.^6,7^ Across these population-based samples from 2 Northern European countries, exposure to close others’ SITB was a significant mediator in the association between sexual orientation and one’s own SITB. The relatively high proportion of the mediation explained (ranging from approximately one-third to one-half) by exposure to close others’ SITB across both samples highlights this experience as a potential mechanism warranting further study. Supplemental analyses showed that these associations held even after controlling for exposure to stigma-related stress. These analyses also revealed differential patterns by sex and sexual orientation, with disparities most pronounced among women. In particular, bisexual women and those who identified their sexual orientation as “something else” or “I don’t know” reported the highest levels of both exposure to others’ SITB and their own SITB. Prior research has consistently shown that bisexual women are at elevated risk for SITB compared with both heterosexual women and lesbians, with acute life stressors—such as job loss, financial hardship, and relationship problems—identified as key contributing factors.^31^ The current study extends this literature by identifying exposure to others’ SITB as an additional potential risk mechanism among bisexual women, warranting further theoretical development as to the reasons underlying the heightened risk in this group.

These findings highlight the need to examine how sexual minority people’s social networks may contribute to exposure to close others’ SITB and SITB transmission. Neither study specified whether exposure to close others’ SITB occurred through peers, family members, or another source, which limits insight into how different social relationships may shape risk. However, prior research has documented SITB transmission within families,^2^ through social contagion in schools and psychiatric settings,^32^ and even at the population level following high-profile suicides.^33^ Future research should explore how identity-related psychosocial factors—such as suicide scripts (ie, cultural and community norms regarding suicide^10,11^), cognitive availability (ie, the conceptualization of suicide as a possible option^34^), and group identification (ie, the degree to which individuals feel a shared sense of identity with their community)—may shape SITB transmission, including among sexual minority people. For example, within more tightly connected networks of sexual minority people, these identity-related psychosocial factors may reinforce SITB normalization and perceived acceptability as a response to shared stigma-related stress, thus increasing SITB.^13^ Understanding how these dynamics operate could then inform tailored interventions to disrupt SITB transmission and refine existing approaches for sexual minority individuals. For example, if future research identifies that community norms regarding the acceptability of SITB contribute to its transmission, existing peer-based interventions with demonstrated effectiveness, such as the school-based Sources of Strength program that aims to diffuse healthy norms and promote coping, may warrant adaptation to incorporate norm-changing strategies that are responsive to the experiences of sexual minority youths.^35^ At the same time, studying how psychosocial factors may influence SITB transmission specifically among sexual minority people has broader implications for the field by offering insights into the mechanisms by which identity-related factors and community dynamics and norms may shape SITB risk transmission pathways in the general population. This knowledge can help researchers identify population-specific risks, refine theoretical models of SITB transmission, and inform the development of interventions.

### Limitations

This study has limitations. First, the relatively low base rate of SITB, particularly suicide attempt, in the population limited the statistical power of the study and precluded subgroup analyses, especially in the SWS. Relatedly, some confidence intervals were wide, likely reflecting uncertainty due to low power. Second, as mentioned previously, neither study investigated the nature of the relationship between the individual and the source of SITB exposure (eg, family member, friend), limiting understanding of how different relational exposures may influence SITB outcomes. Third, the study relied on self-reported survey assessments of SITB and exposure to close others’ SITB with relatively long recall periods (eg, past year), which may have contributed to recall bias. Fourth, while the SWS dataset was longitudinal, it did not include repeated assessment of all variables, and the PLUS dataset used in these analyses was cross-sectional, which limits both causal inference and understanding of the directionality of associations. To overcome these limitations, future research may consider employing intensive prospective data collection methods, such as ecological momentary assessment,^36^ to map the role of exposure to close others’ SITB in the short-term prediction of SITB risk. Fifth, neither study used comprehensive social network methodologies, such as egocentric approaches,^37^ which involve participants reporting detailed characteristics of each member of their social network, or dyadic designs,^38^ which track pairs of peers over time to assess the dynamics of risk transmission. Incorporating such methodologies would enable researchers to examine how network structures, relational dynamics, shared stressors, and perceived norms may uniquely contribute to SITB transmission risk among sexual minority people. Sixth, while the studies were complementary in nature, they used different assessments of SITB exposure and outcomes. Although this multimeasure approach allows for improved replicability, it limited direct comparison. Additionally, Scotland and Sweden are comparable in many respects—both have relatively small, homogeneous populations with relatively high average income. As such, their high levels of social acceptance and gender equity, as reflected in international indexes,^39,40^ may limit the generalizability of findings to countries with different sociopolitical climates, where sexual minority people may face greater stigma-related stress.

## Conclusions

This cohort study of 2 nationally representative populations of young adults in Northern Europe highlights the association of exposure to close others’ SITB with the sexual orientation disparity in SITB. The findings suggest that such exposure partially mediates this disparity. By identifying the association of exposure to others’ SITB with heightened SITB risk among sexual minority young adults, this study underscores the importance of future research examining how identity-related psychosocial factors influence SITB transmission risk to inform intervention and prevention opportunities.

## Notes

Supplement 1. **eTable 1.** Pearson correlations between variables in the Pathways to Longitudinally Understanding Stress (PLUS) cohort **eTable 2.** Pearson correlations between variables in Scottish Wellbeing Study (SWS) cohort **eTable 3.** Group differences in self-injurious thoughts and behaviors (SITB) variables by sex at birth and sexual orientation, Pathways to Longitudinally Understanding Stress (PLUS) cohort **eFigure 1.** Mediation models depicting direct and indirect effects of sexual orientation to suicidal ideation via exposure to close others’ SITB controlling for discrimination, Pathways to Longitudinally Understanding Stress (PLUS) Cohort **eFigure 2.** Mediation models depicting direct and indirect effects of sexual orientation to SITB via exposure to close others’ SITB controlling for victimization, Scottish Wellbeing Study (SWS) Cohort **eFigure 3.** Mediation models depicting direct and indirect effects of sexual orientation to suicidal ideation via exposure to close others’ SITB with the outcome modeled as binary, Pathways to Longitudinally Understanding Stress (PLUS) Cohort

Supplement 2.
Data Sharing Statement
