# Supplementary material for: Sexual Orientation and Exposure to Close Others’ Self-Injurious Thoughts and Behaviors
Source: JAMA Netw Open. 2025 Sep 10;8(9):e2531182. doi: 10.1001/jamanetworkopen.2025.31182 (PMC12423855; doi:10.1001/jamanetworkopen.2025.31182)
Supplement: Supplement 1. — eTable 1. Pearson correlations between variables in the Pathways to Longitudinally Understanding Stress (PLUS) cohort eTable 2. Pearson correlations between variables in Scottish Wellbeing Study (SWS) cohort eTable 3. Group differences in self-injurious thoughts and behaviors (SITB) variables by sex at birth and sexual orientation, Pathways to Longitudinally Understanding Stress (PLUS) cohort eFigure 1. Mediation models depicting direct and indirect effects of sexual orientation to suicidal ideation via exposure to close others’ SITB controlling for discrimination, Pathways to Longitudinally Understanding Stress (PLUS) Cohort eFigure 2. Mediation models depicting direct and indirect effects of sexual orientation to SITB via exposure to close others’ SITB controlling for victimization, Scottish Wellbeing Study (SWS) Cohort eFigure 3. Mediation models depicting direct and indirect effects of sexual orientation to suicidal ideation via exposure to close others’ SITB with the outcome modeled as binary, Pathways to Longitudinally Understanding Stress (PLUS) Cohort [file jamanetwopen-e2531182-s001.pdf]

Supplementary Online Content

Clark KA, Cleare S, Brausch AM, et al. Sexual orientation and exposure to close others' self-injurious thoughts and behaviors. *JAMA Netw Open*. 2025;8(9):e2531182. doi:10.1001/jamanetworkopen.2025.31182

eTable 1. Pearson correlations between variables in the Pathways to Longitudinally Understanding Stress (PLUS) cohort

eTable 2. Pearson correlations between variables in Scottish Wellbeing Study (SWS) cohort

eTable 3. Group differences in self-injurious thoughts and behaviors (SITB) variables by sex at birth and sexual orientation, Pathways to Longitudinally Understanding Stress (PLUS) cohort

eFigure 1. Mediation models depicting direct and indirect effects of sexual orientation to suicidal ideation via exposure to close others' SITB controlling for discrimination, Pathways to Longitudinally Understanding Stress (PLUS) Cohort

eFigure 2. Mediation models depicting direct and indirect effects of sexual orientation to SITB via exposure to close others' SITB controlling for victimization, Scottish Wellbeing Study (SWS) Cohort

eFigure 3. Mediation models depicting direct and indirect effects of sexual orientation to suicidal ideation via exposure to close others' SITB with the outcome modeled as binary, Pathways to Longitudinally Understanding Stress (PLUS) Cohort

This supplementary material has been provided by the authors to give readers additional information about their work.

eTable 1. Pearson correlations between variables in the Pathways to Longitudinally Understanding Stress (PLUS) cohort

	1	2	3	4	5	6	7	8
1. Sexual minority	-							
2. Sex	0.13*	-						
3. Age	-0.15*	-0.13*	-					
4. Discrimination	0.16*	0.06	-0.07*	-				
5. Exposure to close others' suicidal ideation	0.27*	0.14*	-0.14*	0.38*	-			
6. Exposure to close others' suicide attempt	0.19*	0.11*	-0.07*	0.20*	0.41*	-		
7. Exposure to close others' suicide death	0.07	-0.05	0.06*	0.11*	0.18*	0.21*	-	
8. Past-month suicidal ideation	0.19*	0.03	-0.10*	0.39*	0.34*	0.16*	0.15*	-
**Mean**	0.45	0.75	30.15	1.48	4.44	0.32	0.22	3.19
**SD**	0.50	0.43	5.10	0.47	3.18	0.46	0.41	7.00

Note: * $p < 0.05$, SD= standard deviation. Sexual minority: 0=Heterosexual, 1=Sexual minority; Sex = 0=Male, 1=Female; Age=continuous; Discrimination=Everyday Discrimination Scale (EDS); Exposure to close others' suicidal ideation=Suicidal Behavior Exposure (SBX) suicidal communication subscale; Exposure to close others' suicide attempt: 0=No; 1=Yes; Exposure to close others' suicide death: 0=No; 1=Yes; Past-month suicidal ideation: Suicide Ideation Attributes Scale (SIDAS)

© 2025 Clark KA et al. *JAMA Network Open*.

eTable 2. Pearson correlations between variables in Scottish Wellbeing Study (SWS) cohort

	1	2	3	4	5	6	7	8	9	10	11
1. Sexual minority (T1)	-										
2. Sex (T1)	-0.07*	-									
3. Age (T1)	-0.05*	0.02	-								
4. Victimization (T3)	0.10*	0.03	-0.02	-							
5. Exposure to close others' suicide attempt (T2)	0.10*	0.08*	0.01	0.12*	-						
6. Exposure to close others' non-suicidal self-injury (T2)	0.11*	0.04	-0.01	0.11*	0.55*	-					
7. Exposure to close others' suicide death (T2)	0.08*	0.04	0.02	0.07*	0.45*	0.23*	-				
8. Past-year suicidal ideation (T3)	0.16*	-0.01	-0.01	0.15*	0.12*	0.12*	0.11*	-			
9. Past-year non-suicidal self-injury ideation (T3)	0.15*	0.03	-0.08*	0.18*	0.07*	0.13*	0.06*	0.45*	-		
10. Past-year non-suicidal self-injury (T3)	0.09*	0.04	-0.07*	0.15*	0.10*	0.18*	0.04	0.35*	0.64*	-	
11. Past-year suicide attempt (T3)	0.13*	-0.02	-0.03	0.07*	0.04	0.09*	0.05*	0.25*	0.20*	0.20*	-
**Mean**	0.07	0.58	26.94	0.26	0.15	0.15	0.10	0.14	0.09	0.06	0.01
**SD**	0.25	0.49	4.82	0.44	0.36	0.36	0.31	0.35	0.29	0.24	0.12

Note: * $p < 0.05$, SD= standard deviation. Sexual minority: 0=Heterosexual, 1=Sexual minority; Sex = 0=Male, 1=Female; Age = continuous; Variables 4-11 all assessed as 0=No, 1=Yes; T1= 12month follow-up, T2= 24month follow-up, T3 36month follow-up

© 2025 Clark KA et al. *JAMA Network Open*.

eTable 3. Group differences in self-injurious thoughts and behaviors (SITB) variables by sex at birth and sexual orientation, Pathways to Longitudinally Understanding Stress (PLUS) cohort.

		Male				Female			
**Variable**	**Sexual orientation**	**Group N**	**Mean**	**Std**	**p-value[a]**	**Group N**	**Mean**	**Std**	**p-value[a]**
**Exposure to close others' suicidal ideation[b]**	Lesbian or Gay	51	4.29	2.98	0.012	65	5.35	3.26	<.001
	Bisexual	38	4.34	2.62	0.019	293	5.53	3.16	<.001
	Something else/I don't know	11	5.55	3.62	0.005	84	6.08	3.54	<.001
	Heterosexual	196	3.23	2.53	ref	464	3.84	3.06	ref
**Exposure to close others' suicide attempt**	Lesbian or Gay	50	0.26	0.44	0.489	65	0.42	0.50	0.003
	Bisexual	38	0.24	0.43	0.758	293	0.46	0.50	<.001
	Something else/I don't know	11	0.18	0.40	0.798	84	0.46	0.50	<.001
	Heterosexual	196	0.21	0.41	ref	464	0.24	0.43	ref
**Exposure to close others' suicide death**	Lesbian or Gay	50	0.24	0.43	0.770	65	0.22	0.41	0.324
	Bisexual	38	0.26	0.45	0.970	293	0.28	0.45	<.001
	Something else/I don't know	11	0.18	0.41	0.566	84	0.17	0.37	0.987
	Heterosexual	196	0.26	0.44	ref	464	0.17	0.37	ref
**Exposure to close others' suicide attempt/death composite**	Lesbian or Gay	50	0.38	0.49	0.922	65	0.48	0.50	0.033
	Bisexual	38	0.42	0.50	0.573	293	0.56	0.50	<.001
	Something else/I don't know	11	0.36	0.50	0.953	84	0.55	0.50	<.001
	Heterosexual	196	0.37	0.48	ref	464	0.34	0.47	ref
**Past-month suicidal ideation**	Lesbian or Gay	50	3.04	5.98	0.390	65	3.40	7.51	0.105
	Bisexual	38	4.37	7.98	0.049	293	4.70	7.95	<.001
	Something else/I don't know	11	6.09	9.40	0.044	84	6.49	10.20	<.001
	Heterosexual	196	2.19	5.68	ref	464	1.88	5.53	ref

[a] p-values derived from linear regression models (for continuous variables) or logistic regression models (for binary variables)
[b]Exposure to close others' suicidal ideation measured through the Suicidal Behavior Exposure (SBX) suicidal communication subscale

© 2025 Clark KA et al. *JAMA Network Open*.

eFigure 1. Mediation models depicting direct and indirect effects of sexual orientation to suicidal ideation via exposure to close others' SITB controlling for discrimination, Pathways to Longitudinally Understanding Stress (PLUS) Cohort

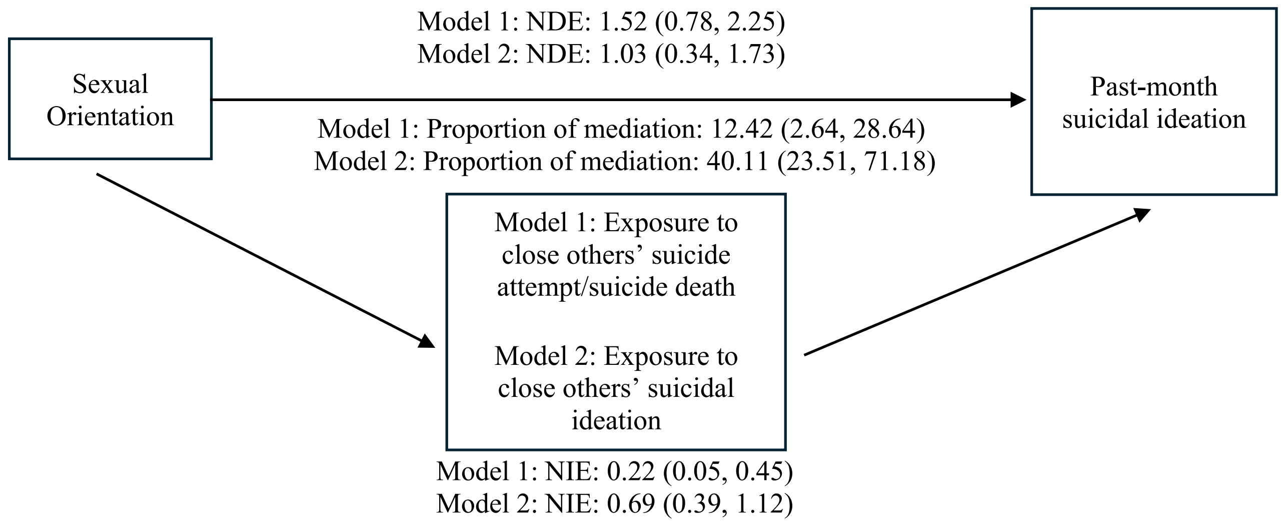

Note: NDE=natural direct effect; NIE=natural indirect effect. NDE and NIE estimates report: β (bias-corrected bootstrap 95% confidence interval). Proportion of mediation report: % mediated (bias-corrected bootstrap 95% confidence interval). Model 1 Mediator: Exposure to close others' suicide attempt/suicide death (exposed = 1; unexposed = 0); Model 2 Mediator: Exposure to close others' suicidal ideation (Suicidal Behavior Exposure [SBX] suicidal communication subscale score); Both Models: Exposure = Sexual orientation (sexual minority = 1; heterosexual = 0), Outcome = Past-month suicidal ideation (continuous Suicidal Ideation Attributes Scale [SIDAS] score). Both models adjusted for age (in years), sex assigned at birth (male, female), and discrimination (Everyday Discrimination Scale [EDS] score).

© 2025 Clark KA et al. *JAMA Network Open*.

eFigure 2. Mediation models depicting direct and indirect effects of sexual orientation to SITB via exposure to close others' SITB controlling for victimization, Scottish Wellbeing Study (SWS) Cohort

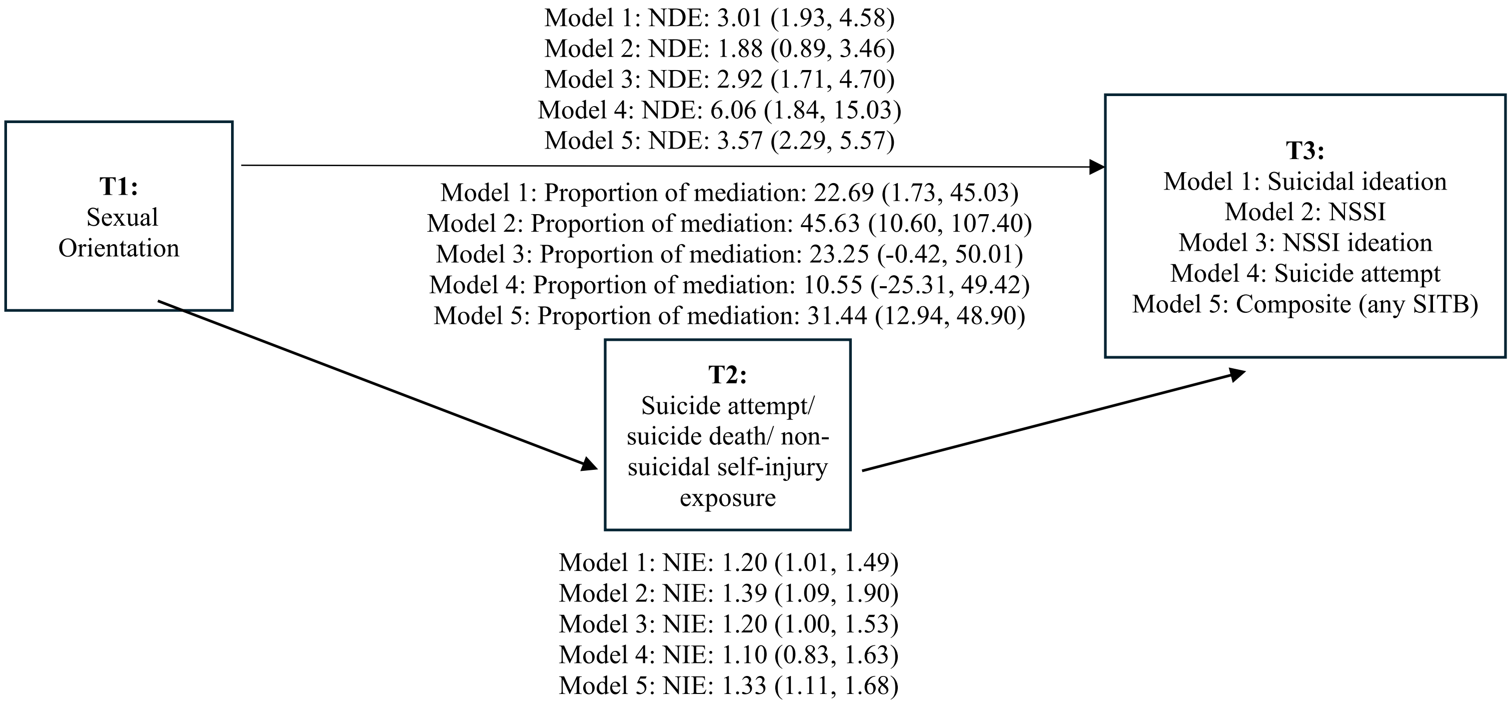

Note: All estimates report: odds ratio (bias-corrected bootstrap 95% confidence interval). NDE=natural direct effect; NIE=natural indirect effect. T1=time 1; T2=time 2 (12-month follow-up); T3=time 3 (24-month follow-up). Model 1 Outcome = past-year suicidal ideation (1 = any; 0 = none); Model 2 Outcome = past-year SITB engagement (1 = any; 0 = none); Model 3 Outcome = past-year SITB ideation (1 = any; 0 = none); Model 4 Outcome = past-year suicide attempt (1 = any; 0 = none); Model 5 Outcome = past-year SITB ideation or behavior (1 = any; 0 = none); All Models: Exposure = Sexual orientation (sexual minority = 1; heterosexual = 0), Mediator = Suicide/SITB exposure (exposed = 1; unexposed = 0). Models adjusted for age (in years), sex assigned at birth (male, female), and victimization (1 = any; 0 = none).

© 2025 Clark KA et al. *JAMA Network Open*.

eFigure 3. Mediation models depicting direct and indirect effects of sexual orientation to suicidal ideation via exposure to close others' SITB with the outcome modeled as binary, Pathways to Longitudinally Understanding Stress (PLUS) Cohort

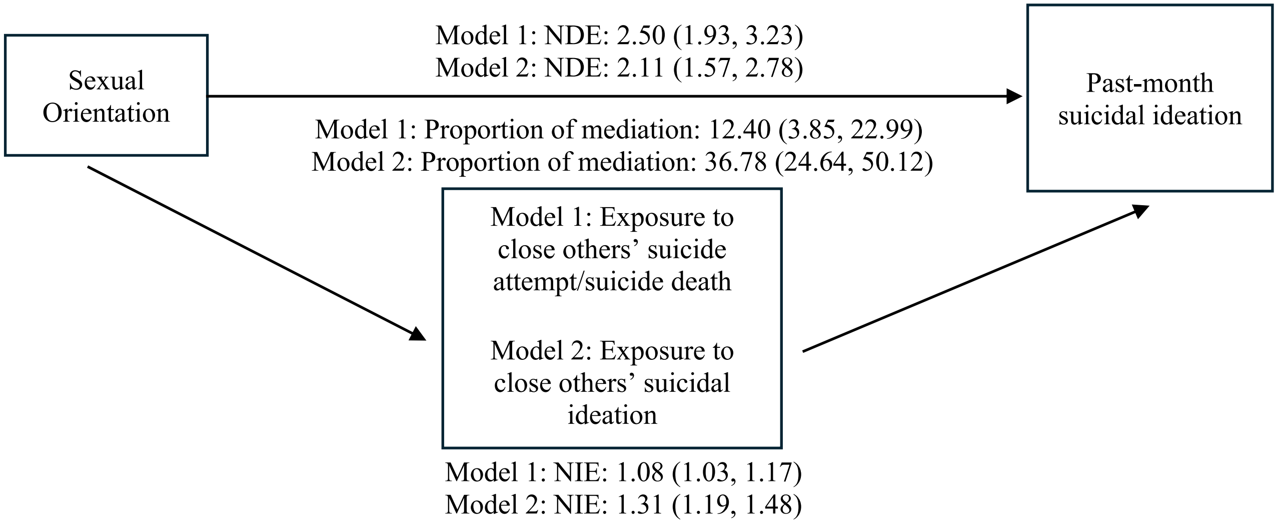

Note: NDE=natural direct effect; NIE=natural indirect effect. NDE and NIE estimates report: odds ratio (bias-corrected bootstrap 95% confidence interval). Proportion of mediation report: % mediated (bias-corrected bootstrap 95% confidence interval). Model 1 Exposure to close others' suicide attempt/suicide death (exposed = 1; unexposed = 0); Model 2 Mediator: Exposure to close others' suicidal ideation (Suicidal Behavior Exposure [SBX] suicidal communication subscale score); Both Models: Exposure = Sexual orientation (sexual minority = 1; heterosexual = 0), Outcome = Past-month suicidal ideation dichotomized based on Suicidal Ideation Attributes Scale [SIDAS] score (1 = any; 0 = none). Both models adjusted for age (in years), sex assigned at birth (male, female), and discrimination (Everyday Discrimination Scale [EDS] score).

© 2025 Clark KA et al. *JAMA Network Open*.
