# Supplementary material for: Sexual Orientation and Exposure to Close Others’ Self-Injurious Thoughts and Behaviors
Source: JAMA Netw Open. 2025 Sep 10;8(9):e2531182. doi: 10.1001/jamanetworkopen.2025.31182 (PMC12423855; doi:10.1001/jamanetworkopen.2025.31182)
Supplement: Supplement 2. — Data Sharing Statement [file jamanetwopen-e2531182-s002.pdf]

Data Sharing Statement

Clark. Sexual Orientation and Exposure to Close Others' Self-Injurious Thoughts and Behaviors. *JAMA Netw Open*. Published September 10, 2025.
doi:10.1001/jamanetworkopen.2025.31182

Data
Data available: No

Additional Information
Explanation for why data not available: PLUS cohort data were produced under the Swedish Statistics Act and the European Union Data Protection Regulation, according to which privacy concerns restrict the availability of personal data for research. Aggregated data can be made available by the authors, subject to ethical vetting.
